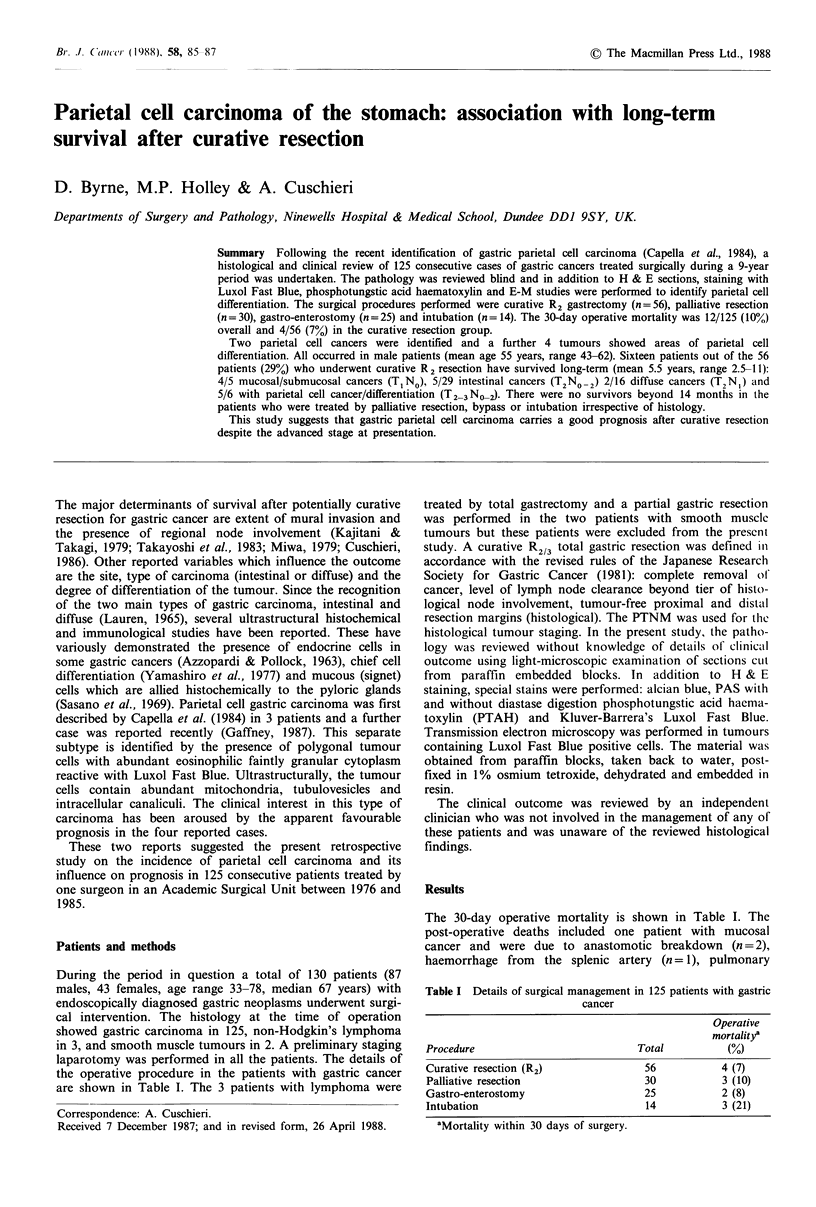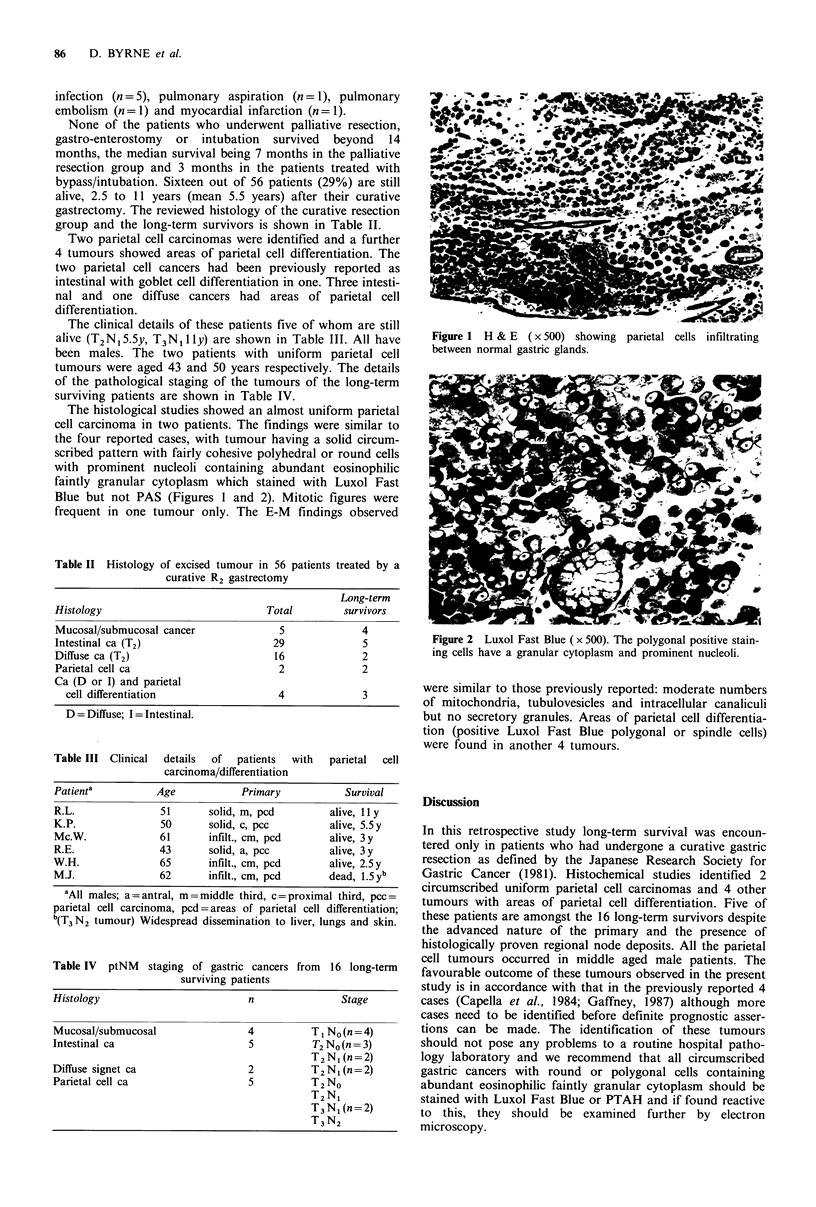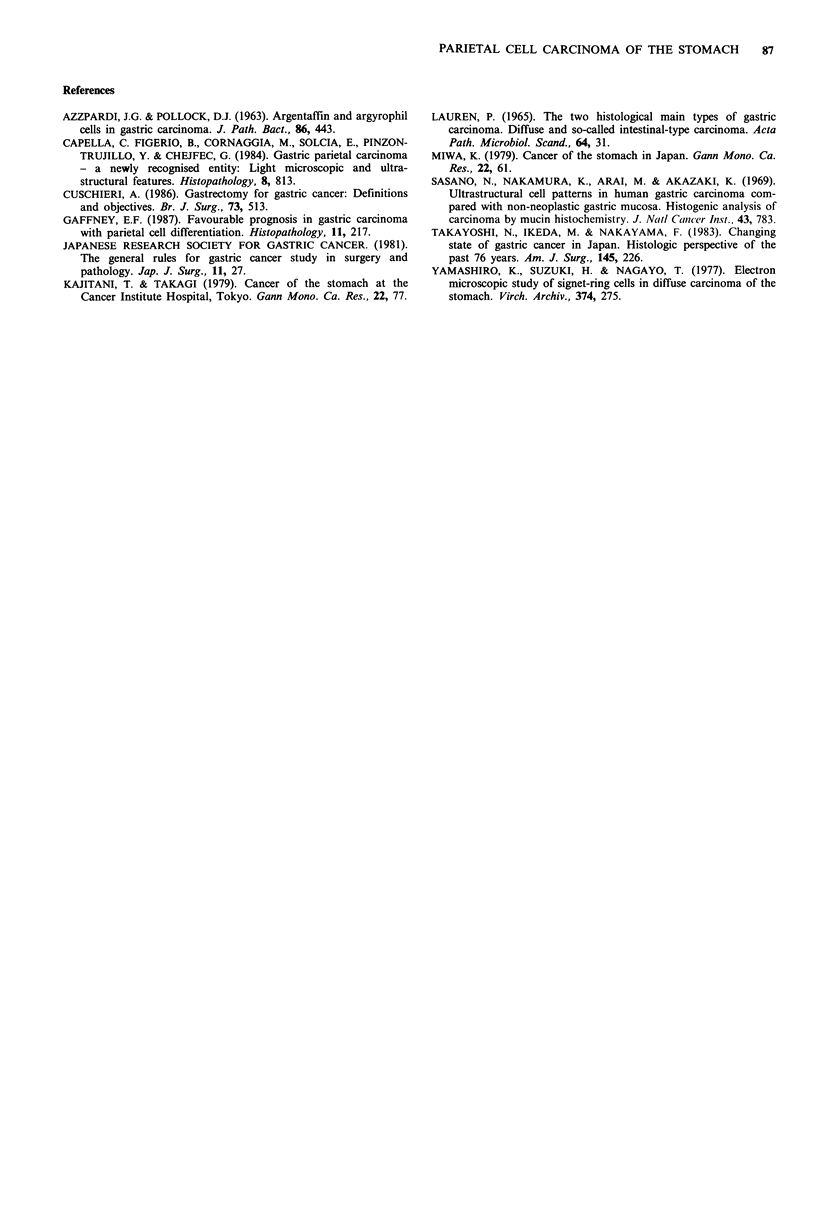# Parietal cell carcinoma of the stomach: association with long-term survival after curative resection.

**DOI:** 10.1038/bjc.1988.168

**Published:** 1988-07

**Authors:** D. Byrne, M. P. Holley, A. Cuschieri

**Affiliations:** Department of Surgery, Ninewells Hospital & Medical School, Dundee, UK.

## Abstract

**Images:**


Br. J. Cancer (1988), 58, 85–87

© The Macmillan Press Ltd., 1988

### Parietal cell carcinoma of the stomach: association with long-term survival after curative resection

D. Byrne, M.P. Holley & A. Cuschieri

*Departments of Surgery and Pathology, Ninewells Hospital & Medical School, Dundee DD1 9SY, UK.*

**Summary** Following the recent identification of gastric parietal cell carcinoma (Capella *et al.*, 1984), a histological and clinical review of 125 consecutive cases of gastric cancers treated surgically during a 9-year period was undertaken. The pathology was reviewed blind and in addition to H & E sections, staining with Luxol Fast Blue, phosphotungstic acid haematoxylin and E-M studies were performed to identify parietal cell differentiation. The surgical procedures performed were curative $R_2$ gastrectomy ($n=56$), palliative resection ($n=30$), gastro-enterostomy ($n=25$) and intubation ($n=14$). The 30-day operative mortality was 12/125 (10%) overall and 4/56 (7%) in the curative resection group.

Two parietal cell cancers were identified and a further 4 tumours showed areas of parietal cell differentiation. All occurred in male patients (mean age 55 years, range 43–62). Sixteen patients out of the 56 patients (29%) who underwent curative $R_2$ resection have survived long-term (mean 5.5 years, range 2.5–11): 4/5 mucosal/submucosal cancers ($T_1N_0$), 5/29 intestinal cancers ($T_2N_{0-2}$) 2/16 diffuse cancers ($T_2N_1$) and 5/6 with parietal cell cancer/differentiation ($T_{2-3}N_{0-2}$). There were no survivors beyond 14 months in the patients who were treated by palliative resection, bypass or intubation irrespective of histology.

This study suggests that gastric parietal cell carcinoma carries a good prognosis after curative resection despite the advanced stage at presentation.

The major determinants of survival after potentially curative resection for gastric cancer are extent of mural invasion and the presence of regional node involvement (Kajitani & Takagi, 1979; Takayoshi *et al.*, 1983; Miwa, 1979; Cuschieri, 1986). Other reported variables which influence the outcome are the site, type of carcinoma (intestinal or diffuse) and the degree of differentiation of the tumour. Since the recognition of the two main types of gastric carcinoma, intestinal and diffuse (Lauren, 1965), several ultrastructural histochemical and immunological studies have been reported. These have variously demonstrated the presence of endocrine cells in some gastric cancers (Azzopardi & Pollock, 1963), chief cell differentiation (Yamashiro *et al.*, 1977) and mucous (signet) cells which are allied histochemically to the pyloric glands (Sasano *et al.*, 1969). Parietal cell gastric carcinoma was first described by Capella *et al.* (1984) in 3 patients and a further case was reported recently (Gaffney, 1987). This separate subtype is identified by the presence of polygonal tumour cells with abundant eosinophilic faintly granular cytoplasm reactive with Luxol Fast Blue. Ultrastructurally, the tumour cells contain abundant mitochondria, tubulovesicles and intracellular canaliculi. The clinical interest in this type of carcinoma has been aroused by the apparent favourable prognosis in the four reported cases.

These two reports suggested the present retrospective study on the incidence of parietal cell carcinoma and its influence on prognosis in 125 consecutive patients treated by one surgeon in an Academic Surgical Unit between 1976 and 1985.

### Patients and methods

During the period in question a total of 130 patients (87 males, 43 females, age range 33–78, median 67 years) with endoscopically diagnosed gastric neoplasms underwent surgical intervention. The histology at the time of operation showed gastric carcinoma in 125, non-Hodgkin's lymphoma in 3, and smooth muscle tumours in 2. A preliminary staging laparotomy was performed in all the patients. The details of the operative procedure in the patients with gastric cancer are shown in Table I. The 3 patients with lymphoma were treated by total gastrectomy and a partial gastric resection was performed in the two patients with smooth muscle tumours but these patients were excluded from the present study. A curative $R_{2/3}$ total gastric resection was defined in accordance with the revised rules of the Japanese Research Society for Gastric Cancer (1981): complete removal of cancer, level of lymph node clearance beyond tier of histological node involvement, tumour-free proximal and distal resection margins (histological). The PTNM was used for the histological tumour staging. In the present study, the pathology was reviewed without knowledge of details of clinical outcome using light-microscopic examination of sections cut from paraffin embedded blocks. In addition to H & E staining, special stains were performed: alcian blue, PAS with and without diastase digestion phosphotungstic acid haematoxylin (PTAH) and Kluver-Barrera's Luxol Fast Blue. Transmission electron microscopy was performed in tumours containing Luxol Fast Blue positive cells. The material was obtained from paraffin blocks, taken back to water, postfixed in 1% osmium tetroxide, dehydrated and embedded in resin.

The clinical outcome was reviewed by an independent clinician who was not involved in the management of any of these patients and was unaware of the reviewed histological findings.

### Results

The 30-day operative mortality is shown in Table I. The post-operative deaths included one patient with mucosal cancer and were due to anastomotic breakdown ($n=2$), haemorrhage from the splenic artery ($n=1$), pulmonary

**Table I** Details of surgical management in 125 patients with gastric cancer

| Procedure | Total | *Operative mortality*[a] (%) |
|---|---|---|
| Curative resection ($R_2$) | 56 | 4 (7) |
| Palliative resection | 30 | 3 (10) |
| Gastro-enterostomy | 25 | 2 (8) |
| Intubation | 14 | 3 (21) |

[a]Mortality within 30 days of surgery.

Correspondence: A. Cuschieri.
Received 7 December 1987; and in revised form, 26 April 1988.

infection ($n=5$), pulmonary aspiration ($n=1$), pulmonary embolism ($n=1$) and myocardial infarction ($n=1$).

None of the patients who underwent palliative resection, gastro-enterostomy or intubation survived beyond 14 months, the median survival being 7 months in the palliative resection group and 3 months in the patients treated with bypass/intubation. Sixteen out of 56 patients (29%) are still alive, 2.5 to 11 years (mean 5.5 years) after their curative gastrectomy. The reviewed histology of the curative resection group and the long-term survivors is shown in Table II.

Two parietal cell carcinomas were identified and a further 4 tumours showed areas of parietal cell differentiation. The two parietal cell cancers had been previously reported as intestinal with goblet cell differentiation in one. Three intestinal and one diffuse cancers had areas of parietal cell differentiation.

The clinical details of these patients five of whom are still alive ($T_2N_1 5.5y$, $T_3N_1 11y$) are shown in Table III. All have been males. The two patients with uniform parietal cell tumours were aged 43 and 50 years respectively. The details of the pathological staging of the tumours of the long-term surviving patients are shown in Table IV.

The histological studies showed an almost uniform parietal cell carcinoma in two patients. The findings were similar to the four reported cases, with tumour having a solid circumscribed pattern with fairly cohesive polyhedral or round cells with prominent nucleoli containing abundant eosinophilic faintly granular cytoplasm which stained with Luxol Fast Blue but not PAS (Figures 1 and 2). Mitotic figures were frequent in one tumour only. The E-M findings observed

**Figure 1** H & E (×500) showing parietal cells infiltrating between normal gastric glands.

**Figure 2** Luxol Fast Blue (×500). The polygonal positive staining cells have a granular cytoplasm and prominent nucleoli.

were similar to those previously reported: moderate numbers of mitochondria, tubulovesicles and intracellular canaliculi but no secretory granules. Areas of parietal cell differentiation (positive Luxol Fast Blue polygonal or spindle cells) were found in another 4 tumours.

**Table II** Histology of excised tumour in 56 patients treated by a curative $R_2$ gastrectomy

| *Histology* | *Total* | *Long-term survivors* |
|---|---|---|
| Mucosal/submucosal cancer | 5 | 4 |
| Intestinal ca ($T_2$) | 29 | 5 |
| Diffuse ca ($T_2$) | 16 | 2 |
| Parietal cell ca | 2 | 2 |
| Ca (D or I) and parietal cell differentiation | 4 | 3 |

D = Diffuse; I = Intestinal.

**Table III** Clinical details of patients with parietal cell carcinoma/differentiation

| *Patient*[a] | *Age* | *Primary* | *Survival* |
|---|---|---|---|
| R.L. | 51 | solid, m, pcd | alive, 11 y |
| K.P. | 50 | solid, c, pcc | alive, 5.5 y |
| Mc.W. | 61 | infilt., cm, pcd | alive, 3 y |
| R.E. | 43 | solid, a, pcc | alive, 3 y |
| W.H. | 65 | infilt., cm, pcd | alive, 2.5 y |
| M.J. | 62 | infilt., cm, pcd | dead, 1.5 y[b] |

[a]All males; a = antral, m = middle third, c = proximal third, pcc = parietal cell carcinoma, pcd = areas of parietal cell differentiation; [b]($T_3N_2$ tumour) Widespread dissemination to liver, lungs and skin.

**Table IV** ptNM staging of gastric cancers from 16 long-term surviving patients

| *Histology* | *n* | *Stage* |
|---|---|---|
| Mucosal/submucosal | 4 | $T_1N_0 (n=4)$ |
| Intestinal ca | 5 | $T_2N_0 (n=3)$ |
| | | $T_2N_1 (n=2)$ |
| Diffuse signet ca | 2 | $T_2N_1 (n=2)$ |
| Parietal cell ca | 5 | $T_2N_0$ |
| | | $T_2N_1$ |
| | | $T_3N_1 (n=2)$ |
| | | $T_3N_2$ |

### Discussion

In this retrospective study long-term survival was encountered only in patients who had undergone a curative gastric resection as defined by the Japanese Research Society for Gastric Cancer (1981). Histochemical studies identified 2 circumscribed uniform parietal cell carcinomas and 4 other tumours with areas of parietal cell differentiation. Five of these patients are amongst the 16 long-term survivors despite the advanced nature of the primary and the presence of histologically proven regional node deposits. All the parietal cell tumours occurred in middle aged male patients. The favourable outcome of these tumours observed in the present study is in accordance with that in the previously reported 4 cases (Capella et al., 1984; Gaffney, 1987) although more cases need to be identified before definite prognostic assertions can be made. The identification of these tumours should not pose any problems to a routine hospital pathology laboratory and we recommend that all circumscribed gastric cancers with round or polygonal cells containing abundant eosinophilic faintly granular cytoplasm should be stained with Luxol Fast Blue or PTAH and if found reactive to this, they should be examined further by electron microscopy.

### References

AZZPARDI, J.G. & POLLOCK, D.J. (1963). Argentaffin and argyrophil cells in gastric carcinoma. *J. Path. Bact.*, **86**, 443.

CAPELLA, C. FIGERIO, B., CORNAGGIA, M., SOLCIA, E., PINZON-TRUJILLO, Y. & CHEJFEC, G. (1984). Gastric parietal carcinoma – a newly recognised entity: Light microscopic and ultrastructural features. *Histopathology*, **8**, 813.

CUSCHIERI, A. (1986). Gastrectomy for gastric cancer: Definitions and objectives. *Br. J. Surg.*, **73**, 513.

GAFFNEY, E.F. (1987). Favourable prognosis in gastric carcinoma with parietal cell differentiation. *Histopathology*, **11**, 217.

JAPANESE RESEARCH SOCIETY FOR GASTRIC CANCER. (1981). The general rules for gastric cancer study in surgery and pathology. *Jap. J. Surg.*, **11**, 27.

KAJITANI, T. & TAKAGI (1979). Cancer of the stomach at the Cancer Institute Hospital, Tokyo. *Gann Mono. Ca. Res.*, **22**, 77.

LAUREN, P. (1965). The two histological main types of gastric carcinoma. Diffuse and so-called intestinal-type carcinoma. *Acta Path. Microbiol. Scand.*, **64**, 31.

MIWA, K. (1979). Cancer of the stomach in Japan. *Gann Mono. Ca. Res.*, **22**, 61.

SASANO, N., NAKAMURA, K., ARAI, M. & AKAZAKI, K. (1969). Ultrastructural cell patterns in human gastric carcinoma compared with non-neoplastic gastric mucosa. Histogenic analysis of carcinoma by mucin histochemistry. *J. Natl Cancer Inst.*, **43**, 783.

TAKAYOSHI, N., IKEDA, M. & NAKAYAMA, F. (1983). Changing state of gastric cancer in Japan. Histologic perspective of the past 76 years. *Am. J. Surg.*, **145**, 226.

YAMASHIRO, K., SUZUKI, H. & NAGAYO, T. (1977). Electron microscopic study of signet-ring cells in diffuse carcinoma of the stomach. *Virch. Archiv.*, **374**, 275.